# CMTM6-Deficient Monocytes in ANCA-Associated Vasculitis Fail to Present the Immune Checkpoint PD-L1

**DOI:** 10.3389/fimmu.2021.673912

**Published:** 2021-05-24

**Authors:** Markus Zeisbrich, Nina Chevalier, Bettina Sehnert, Marta Rizzi, Nils Venhoff, Jens Thiel, Reinhard E. Voll

**Affiliations:** Department of Rheumatology and Clinical Immunology, Medical Center – University of Freiburg, University of Freiburg, Freiburg, Germany

**Keywords:** ANCA vasculitis, PD-L1, macrophages, lysosomes, immune checkpoint, vasculitis < rheumatic diseases, monocytes

## Abstract

**Objectives:**

ANCA-associated vasculitides (AAV) affect small- and medium-sized blood vessels. In active disease, vessel wall infiltrates are mainly composed of monocytes and macrophages. Immune checkpoint molecules are crucial for the maintenance of self-tolerance and the prevention of autoimmune diseases. After checkpoint inhibitor therapy, the development of autoimmune vasculitis has been observed. However, defects of immune checkpoint molecules in AAV patients have not been identified yet.

**Methods:**

Monocytes and monocyte-derived macrophages from AAV patients and healthy age-matched controls were tested for surface expression of immunoinhibitory checkpoint programmed cell death ligand-1 (PD-L1). Using *in vitro* co-culture approaches, the effect of monocyte PD-L1 expression on CD4^+^ T cell activation and proliferation was tested.

**Results:**

Monocytes from AAV patients displayed lower PD-L1 expression and a defective PD-L1 presentation upon activation, an effect that was correlated with disease activity. Lower PD-L1 expression was due to increased lysosomal degradation of PD-L1 in AAV monocytes. We identified a reduced expression of CMTM6, a protein protecting PD-L1 from lysosomal breakdown, as the underlying molecular defect. PD-L1^low^ AAV monocytes showed increased stimulatory capacity and induced T cell activation and proliferation. Inhibiting lysosomal function corrected this phenotype by increasing PD-L1, thus normalizing the pro-stimulatory behavior of AAV monocytes.

**Conclusions:**

This study identifies a defect of the immunoinhibitory checkpoint PD-L1 in monocytes from patients with AAV. Low expression of CMTM6 results in enhanced lysosomal degradation of PD-L1, thus providing insufficient negative signaling to T cells. Correcting this defect by targeting lysosomal function may represent a novel strategy to treat AAV.

## Introduction

Antineutrophil cytoplasmic antibody (ANCA)–associated vasculitides (AAV) are characterized by necrotizing inflammation of small blood vessels and the presence of ANCA with specificity for proteinase-3 (PR3) or myeloperoxidase (MPO). Frequent target tissues are the respiratory tract, kidneys, skin, and peripheral nerves. Immunopathologically, AAV present with an absence or paucity of immunoglobulin and complement deposition in affected vessels. Early vascular lesions of AAV consist of neutrophils with admixed monocytes. Within days, the initial inflammatory lesion is replaced by inflammation with a predominance of monocytes and macrophages (1). Also in renal biopsies, monocytes and macrophages are the predominant cells in glomeruli of patients with AAV (2, 3).

In AAV, systemic monocyte activation has been observed and their activation state persisted during remission (4–6). Transmembrane receptors that facilitate extracellular matrix adhesion are upregulated on AAV monocytes indicating an increased ability to interact with vascular endothelium (7). Monocytes also express antigens recognized by ANCA, and stimulation *in vitro* with ANCA promoted cytokine and reactive oxygen species (ROS) production (8, 9). Moreover, AAV monocytes recognize neutrophil extracellular traps (NETs) to upregulate the alarmin S100A9, which results in the induction of metalloproteinase-9 and enables monocytes to invade into the extracellular matrix (10).

Immune checkpoint molecules are crucial for the maintenance of self-tolerance. Programmed death ligand-1 (PD-L1) is an inhibitory checkpoint molecule that counterbalances the overwhelming activation of the immune system. PD-L1 is expressed on antigen-presenting cells (monocytes, macrophages, and dendritic cells) and endothelial cells (11). By engaging its ligand programmed death-1 (PD-1) on T cells, TCR- and CD28-mediated activation cascades are down-regulated. In animal studies, disruption of PD-L1 signals has been associated with inflammatory disease (12–14). In line with this, a defect in the PD-1/PD-L1 axis was reported in large vessel vasculitis (15, 16). In cancer immunotherapy, the use of checkpoint inhibitors that block the PD-1/PD-L1 pathway has proven to be very effective (17–19). In parallel, increasing reports of immune-related adverse events were noted (irAEs) (20). In fact, several forms of vasculitis have been reported to develop or re-activate after checkpoint inhibitor therapy, including AAV (21–24).

Here, we report that monocytes from AAV patients express low levels of the inhibitory checkpoint PD-L1. Patient-derived cells showed a defect in presenting PD-L1 in response to inflammatory stimuli, thus leading to increased activation of T cells in co-culture experiments. Mechanistically, inhibiting lysosome function in monocytes from AAV patients corrected their hyperstimulatory phenotype by upregulating PD-L1, indicating that increased lysosomal degradation of PD-L1 occurs in AAV monocytes. In line with this, AAV monocytes display a lower expression of chemokine-like factor-like MARVEL transmembrane domain containing family member 6 (CMTM6), a protein protecting PD-L1 from lysosomal breakdown (25, 26).

This study establishes a link between autoimmune small-vessel vasculitis and immunoinhibitory checkpoint deficiency, highlighting the potential role of uncontrolled innate immunity in promoting the development of autoimmune disease.

## Materials and Methods

### Patients and Controls

The study population included 26 AAV patients classified as granulomatosis with polyangiitis (GPA, n=21) or microscopic polyangiitis (MPA, n=5) as defined by the Chapel Hill Consensus Conference nomenclature (27) and 29 age-matched healthy controls. All AAV patients tested positive for ANCA by indirect immunofluorescence and PR3 or MPO-ELISA and were enrolled between July 2019 and July 2020. Besides, seven RA patients fulfilling the 2010 EULAR/ACR diagnostic criteria were enrolled. Patient blood samples were provided by the Imm-Rheum Biobank of the Department of Rheumatology and Clinical Immunology, University Medical Center Freiburg. Clinical characteristics are given in **Table 1**. Demographically matched healthy individuals were obtained from the Institute for Cell and Gene Therapy at the University Medical Center Freiburg. They had no history of autoimmune disease, cancer, chronic viral infection, or any other inflammatory syndrome. The study was approved by the Institutional Review Board (Ek 218/20, Ek 383/19). Active disease was defined as new onset or recurrence of symptoms that are associated with AAV combined with an increase in C-reactive protein (CRP), which was not explained by an infection, and a Birmingham Vasculitis Activity Score (BVAS) ≥ 1 (28).

**Table 1:** Clinical characteristics of patients and controls.

	AAV	HC	RA
**ANCA-pos. patients** (n)	26	20	7
Proteinase 3 +	19 (73%)		
Myeloperoxidase +	7 (27%)		
**Seropositive** (RF and anti-CCP)			7 (100%)
**Sex**			
Female	17 (65%)	14 (70%)	5 (71%)
Male	9 (35%)	6 (30%)	2 (29%)
**Age** (years, mean ± SD)	61.7 ± 3.4	56,2 ± 5.2	68.2 ± 6.4
**Disease duration** (years, mean ± SD)	6.4 ± 1.3		
**Mean CRP** (mg/l, mean ± SD)	12.6 ± 2.8		7.3 ± 3.4
**DAS28-CRP**			2.5 ± 0.2
**Organ involvement**			
Ear, nose, and throat (ENT)	17 (66%)		
Lungs	18 (69%)		
Kidneys	17 (66%)		
Skin	5 (19%)		
Joints	8 (31%)		
Peripheral nerves	7 (27%)		
**Medication at blood drawing**			
untreated	2 (8%)		0 (0%)
Prednisone	17 (65%)		1 (14%)
Prednisone dose (median ± SD)	4.5 mg ± 4.1		0.2 mg ± 0.1
Rituximab	12 (46%)		0 (0%)
Methotrexate	5 (19%)		5 (71%)
Azathioprin	4 (15%)		0 (0%)
Cyclophosphamide	2 (8%)		0 (0%)
Leflunomide	2 (8%)		0 (0%)
Mycophenolat mofetil	2 (8%)		0 (0%)
Hydroxychloroquine	1 (4%)		2 (29%)

### PR3- and MPO-ELISA

The ANCA staining pattern (cytoplasmatic or perinuclear) was assessed by indirect immunofluorescence. ANCA specificity for proteinase 3 (PR3, Organtec) or myeloperoxidase (MPO, Euroimmun) was measured by enzyme-linked immunosorbent assay (ELISA) and interpreted according to the manufacturers’ reference ranges with the upper limit of the normal of <10 U/ml for PR3 and <20 U/ml for MPO.

### Cells and Culture

Peripheral blood mononuclear cells (PBMCs) were isolated from the peripheral blood by density gradient centrifugation with Lymphoprep (anprotec). Monocytes were isolated using EasySep Human Monocyte Enrichment Kit without CD16 Depletion (Stemcell Technologies) and stimulated with IFNγ (100 IU/ml; PeproTech) or TNFα (5 ng/ml; BioLegend) for 24 hours. To inhibit lysosomal function, cells were treated with Bafilomycin A1 (20 nM; Cayman Chemicals) or chloroquine (10 μM; Sigma-Aldrich). For additional experiments, hydrocortisone (Sigma-Aldrich) was added or monocytes were pre-treated with TNFα (5ng/ml) for 30 minutes and stimulated with 5 μg/ml anti-PR3 (Santa Cruz Biotechnology) or anti-MPO (Miltenyi Biotec).

In co-culture experiments, monocytes were pretreated with IFNγ (100 IU/ml; PeproTech) for 24 hours and then co-cultured with CD4^+^ T cells in a ratio of 1:3 (400,000 monocytes to 1.2 million T cells). CD4^+^ T cells were derived from different HLA-mismatched healthy donors and were distributed equally throughout experiments in order to assess the T cell response based on the difference of the monocyte population. To assess early T cell activation, CD4^+^CD25^+^ T cells were quantified by flow cytometry after 48 hours of co-culture. To assess T cell proliferation, CD4^+^ T cells were labeled with carboxyfluorescein succinimidyl ester (CFSE) and co-cultured with monocytes for 5 days. Proliferation rates were analyzed by CFSE dilution. Monocyte-derived macrophages were differentiated in RPMI 1640 medium (Life technologies) supplemented with 10% FBS (Gibco) and 20 ng/ml M-CSF (BioLegend) or 20 ng/ml GM-CSF (BioLegend) for 6 days as reported previously (29). Cells were detached using StemPro Accutase Cell Dissociation (Life Technologies, Thermo Fisher).

### Quantitative RT-PCR

Total RNA was extracted with Quick-RNA Microprep Kit (Zymo Research), cDNA was reverse transcribed using the High-Capacity cDNA Reverse Transcription Kit (Thermo Fisher Scientific). Gene expression was determined using SYBR Select Master Mix (Applied Biosystems) on an OneStepPlus RT-PCR machine (Applied Biosystems). Used primers are listed below (**Table 2**). Gene transcript numbers were adjusted relative to β-actin transcripts.

**Table 2:** Primer list.

Gene	Forward primer	Reverse primer
β-actin	GATCATTGCTCCTCCTGAGC	CGTCATACTCCTGCTTGCTG
PD-L1	TGGCATTTGCTGAACGCATTT	TGCAGCCAGGTCTAATTGTTTT
CMTM6	TTTCCACACATGACAGGACTTC	GGCTTCAGCCCTAGTGGTAT

### Flow Cytometry

For intracellular staining, cells were permeabilized using the Cytofix/Cytoperm Kit (BD Biosciences). Surface staining and intracellular protein evaluation were done using BD LSR Fortessa. Data were analyzed with FlowJo software (Tree Star). The following antibodies or stainings were used: CMTM6 (biorbyt), PD-L1, PD-L2, CD80, CD86, CD4, CD25 (all BioLegend), and CFSE (Molecular Probes).

### Statistical Analysis

Statistical significance was assessed after data sets were tested for normal Gaussian distribution by D’Agostino normality testing. For data sets with normal distribution, parametric testing was applied (unpaired t-test and paired t-test). For other data sets, non-parametric testing was applied (Mann-Whitney test, Wilcoxon test, Kruskal-Wallis test with Dunn’s multiple comparisons test, and Spearman correlation). All data were analyzed by Prism V.9.1.0 (GraphPad).

## Results

### Reduced Frequency of PD-L1+ Monocytes in Peripheral Blood of AAV Patients

**Figure 1:**
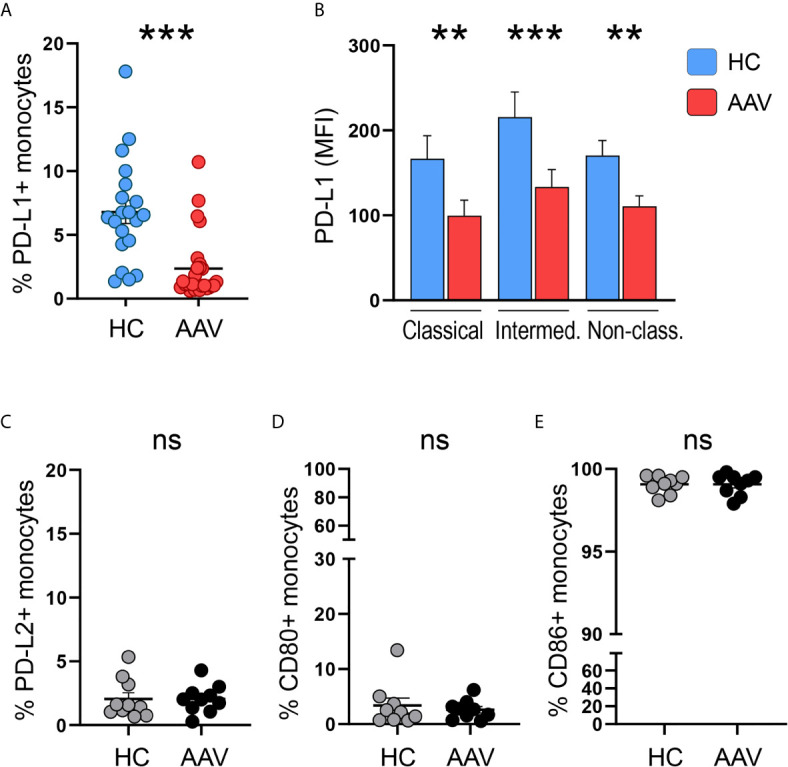
Less PD-L1+ monocytes in patients with AAV. **(A)** Percentages of PD-L1+ monocytes in HC (n=20) and AAV patients (n=26). **(B)** Surface expression of PD-L1 (MFI) on classical (CD14+CD16-), intermediate (CD14+CD16+), and non-classical monocytes (CD14dim CD16++) in healthy control donors (n=20) and AAV patients (n=26). **(C–E)** Percentages of PD-L2+, CD80+, and CD86+ monocytes in HC and AAV patients (n=9-10 each cohort). Mann-Whitney **(A, B, D)** test or unpaired t-test **(C, E)** were applied. **P<0.01; ***P<0.001. Bar graph shows mean ± SEM. AAV, ANCA-associated vasculitis; HC, healthy control donors; MFI, mean fluorescence intensity; PD-L1, Programmed death-ligand 1; ns, statistically not significant.

Monocytes from patients with PR3- or MPO-ANCA-positive AAV were tested for surface expression of the immune checkpoint molecule PD-L1. The frequency of PD-L1+ monocytes in AAV patients was reduced compared to cells from healthy control (HC) donors (**Figure 1A**). A reduction of PD-L1 expression on AAV monocytes was observed across all monocyte subsets (**Figure 1B**; for subset identification see **Supplementary Figure 1**). To evaluate whether this reduction in PD-L1 expression is associated with defects in the expression of other inhibitory or stimulatory molecules, we tested the surface expression of PD-L2, CD80, and CD86. While the inhibitory PD-L2 and the stimulatory CD80 were expressed only weakly, almost all monocytes expressed CD86. Overall, no differences between HC and AAV patients were detected (**Figures 1C–E**). Together, these data demonstrate that patient-derived monocytes have a defect in the expression of PD-L1.

### AAV Monocytes Display a Defect in PD-L1 Presentation Upon Activation

Monocytes acquire PD-L1 expression through extracellular stimuli, e.g. when entering tissue sites and through cytokine stimulation. Analog to tissue infiltration, the culture of monocytes on tissue culture plastic alone is enough to induce PD-L1 expression (30). Still, the strongest known inducer of PD-L1 is IFNγ (31, 32). We tested for PD-L1 induction on monocytes from HC and AAV patients after 24 hours of *in vitro* culture alone or with additional stimulation by IFNγ. As a control, we used monocytes from patients with another chronic inflammatory disease, rheumatoid arthritis (RA), to assess the impact of chronic inflammation on PD-L1 expression.

**Figure 2:**
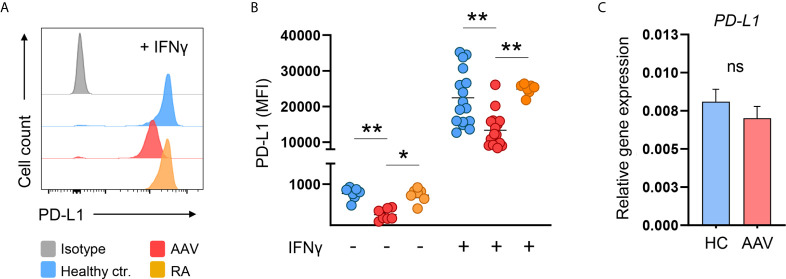
AAV monocytes fail to upregulate PD-L1. **(A)** Representative histograms and **(B)** summarizing scatter plot of PD-L1 surface expression of monocytes left untreated for 24 hours (n=7 each group) or after stimulation with IFNγ for 24 hours (100 IU/ml; HC n=21, AAV n=26, and RA n=7). **(C)** Gene expression of *PD-L1* in monocytes from HC and AAV patients (n=14 each group) after stimulation with IFNγ for 24 hours measured by RT-PCR, relative to housekeeping gene β-actin. Kruskal-Wallis test with Dunn’s multiple comparisons test **(B)** and Mann-Whitney test **(C)** were applied. *P<0.05; **P<0.01. Bar graphs show mean ± SEM. AAV, ANCA-associated vasculitis; HC, healthy control donors; MFI, mean fluorescence intensity; PD-L1, Programmed death-ligand 1; RA, Rheumatoid arthritis; ANCA, Anti-neutrophil cytoplasmic antibodies; ns, statistically not significant.

When left untreated for 24 hours, induction of PD-L1 was weaker in monocytes from AAV patients compared to monocytes from HC and RA patients. After 24 hours of stimulation, IFNγ potently induced PD-L1 expression. Again, monocytes from AAV patients displayed less PD-L1 than HC and RA monocytes. In general, control monocytes from RA patients did not differ from HC monocytes in their PD-L1 expression (**Figures 2A, B**). We further tested TNFα as this cytokine was reported to have at least some effect on PD-L1 expression (31, 32). Overall, its effect was rather weak compared to IFNγ, and also in this set of experiments AAV monocytes failed to properly upregulate PD-L1 (**Supplementary Figure 2**).

The defect in PD-L1 protein presentation of AAV monocytes was not accompanied by a reduction of *PD-L1* mRNA on the transcriptional level (**Figure 2C**). Thus, a global defect in enhancing PD-L1 expression upon activation was detected in AAV monocytes that appeared to be regulated at the protein level.

### Current Medication of AAV Patients Is Not Associated With PD-L1 Expression

As the majority of AAV patients received medical treatment (**Table 1**), we performed a subgroup analysis and examined PD-L1 expression of AAV patients depending on their medication.

**Figure 3:**
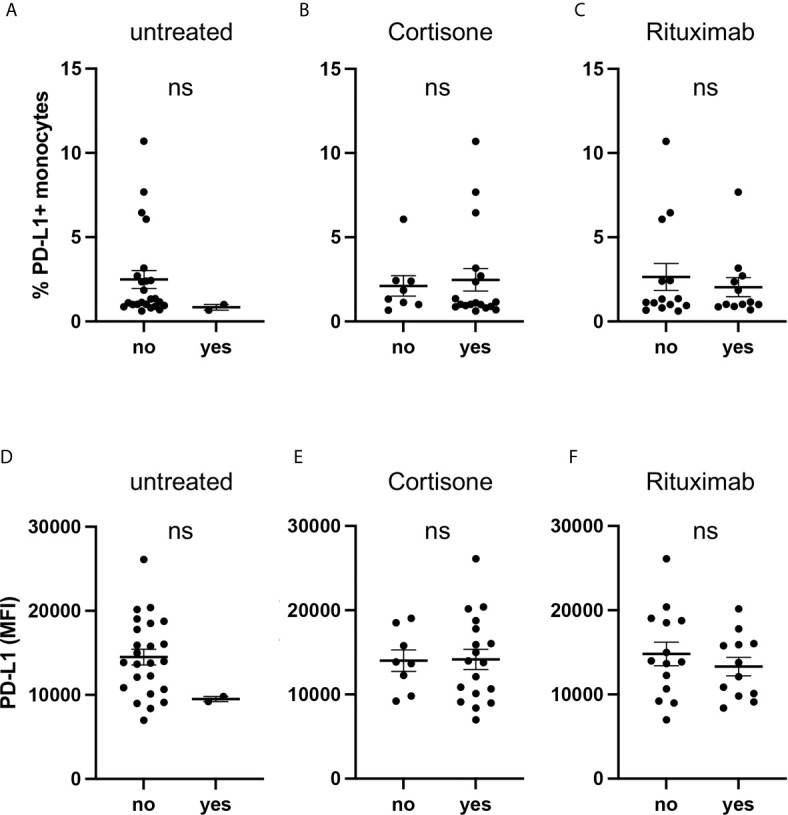
Current medication of AAV patients is not associated with PD-L1 expression. Percentages of PD-L1+ monocytes **(A–C)** and PD-L1 expression of monocytes stimulated with IFNγ for 24 hours **(D–F)** from AAV patients subgrouped depending on their medication. Mann-Whitney test **(A–D)** and unpaired t-test **(E, F)** were applied. MFI, mean fluorescence intensity; PD- L1, Programmed death-ligand 1; ns, statistically not significant.

PD-L1 was low on monocytes from untreated patients on circulating cells (**Figure 3A**) and after activation with IFNγ (**Figure 3D**) but due to the low sample size (n=2) no statistical conclusion can be drawn. We further analyzed subgroups based on cortisone intake (**Figures 3B, E**) and rituximab infusion (**Figures 3C, F**) and observed no relevant effect.

Glucocorticoids (GC) have pleiotropic effects and can act directly on monocytes. We, therefore, treated GC-naïve monocytes with hydrocortisone in different concentrations, either in the presence or absence of additional IFNγ. In summary, hydrocortisone did not alter PD-L1 expression (**Supplementary Figure 3**).

These data indicate that the medication of AAV patients did not bias their monocytes towards low PD-L1 expression.

### PD-L1 Expression on Monocytes Correlates With Disease Activity Markers

We hypothesized that the reduced frequency of PD-L1+ monocytes in the peripheral blood of AAV patients and the induced PD-L1 expression after cell activation are correlated with disease activity.

The low frequency of circulating PD-L1+ monocytes was correlated with high ANCA titers in AAV patients (**Supplementary Figure 4A**), while no correlation was observed between C-reactive protein (CRP) serum concentrations, blood monocyte counts, and renal organ involvement of AAV with the frequency of PD-L1+ monocytes (**Supplementary Figures 4B, C, E**). Although there was a trend for patients with active disease to show lower frequencies of PD-L1+ monocytes, it did not reach statistical significance (**Supplementary Figure 4D**).

**Figure 4:**
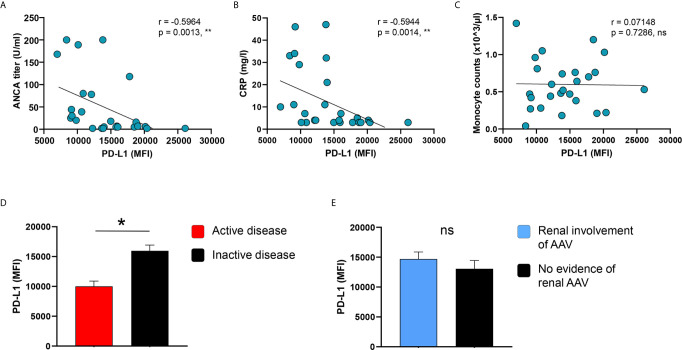
PD-L1 induction on monocytes correlates with disease markers and activity. **(A–C)** Correlation of IFNγ-induced PD-L1 expression with ANCA titers, serum levels of CRP, and blood monocyte counts in patients with AAV (n=26). IFNγ-induced PD-L1 expression on monocytes compared between AAV patients with **(D)** either active or inactive disease (active n=8, inactive n=18) and **(E)** with or without renal AAV (renal AAV n=17, without renal AAV n=9). Spearman correlation **(A–C)** and unpaired t-test **(D, E)** were applied. *P<0.05; **P<0.01. Bar graph shows mean ± SEM. AAV, ANCA-associated vasculitis; HC, healthy control donors; MFI, mean fluorescence intensity; PD-L1, Programmed death-ligand 1; ANCA, Anti-neutrophil cytoplasmic antibodies; CRP, C-reactive protein; ns, statistically not significant.

IFNγ-induced PD-L1 expression correlated inversely with ANCA titers as well as CRP serum concentrations of AAV patients (**Figures 4A, B**), while blood monocyte counts did not correlate with PD-L1 (**Figure 4C**). Moreover, PD-L1 expression after stimulation with IFNγ was lower in patients with active disease (**Figure 4D**) but similar between patients with or without kidney involvement of AAV (**Figure 4E**).

As high ANCA titers correlated with low PD-L1 expression, we tested whether direct stimulation of monocytes with anti-PR3 or anti-MPO antibodies could downregulate PD-L1. Short-term treatment (4 hours) did not alter PD-L1 expression in healthy or AAV monocytes (**Supplementary Figures 5A, B**). After 24 hours, anti-MPO antibodies did not affect PD-L1 expression, while there was a trend for anti-PR3 antibodies to upregulated PD-L1 at least in some of the healthy and patient samples tested (**Supplementary Figures 5C, D**). Also, adding anti-PR3 or anti-MPO antibodies when cells are stimulated with IFNγ did not change PD-L1 levels (**Supplementary Figure 6**).

These data indicate that lower surface presentation of PD-L1 on monocytes is associated with high ANCA titers and disease activity. This effect is not attributed to direct effects of ANCA antibodies on PD-L1.

### Blocking Lysosomal Function Restores PD-L1 Expression in AAV Monocytes That Have Low Expression of CMTM6

**Figure 5:**
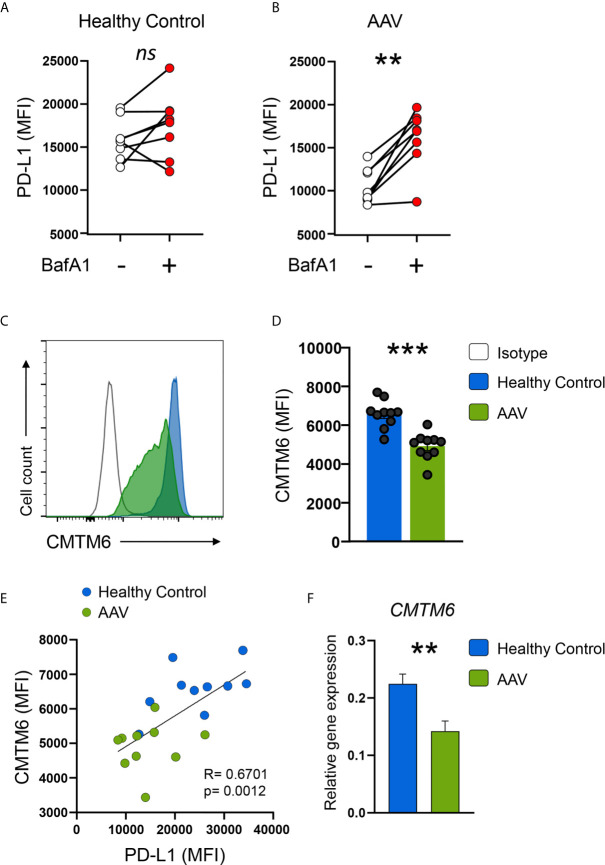
AAV monocytes are deficient for CMTM6, blocking lysosomal function restores capacity to upregulate PD-L1. **(A, B)** Monocytes from either HC **(E)** or AAV patients **(F)** were stimulated with IFNγ for 24 hours in the presence or absence of the lysosomal inhibitor BafA1 (20nM). Monocytes not treated with BafA1 inhibitor were treated with the corresponding vehicle. **(C)** Representative histograms of CMTM6 protein expression in IFNγ-stimulated (24 hours) monocytes from HC and AAV patients. **(D)** Summarizing scatter dot plot showing results from 10 experiments. **(E)** Correlation of PD-L1 expression with CMTM6 in IFNγ-stimulated monocytes in HC and AAV patients (each group n=10). **(F)** Gene expression of *CMTM6* in monocytes from HC and AAV patients after stimulation with IFNγ for 24 hours measured by RT-PCR, relative to housekeeping gene β-actin. Paired t-test **(A)**, Wilcoxon test **(B)**, unpaired t-test **(D)**, Spearman correlation **(E)**, and Mann-Whitney test **(F)** were applied. ***P<0.01; ***P<0.001. Bar graph shows mean ± SEM. AAV, ANCA-associated vasculitis; HC, healthy control donors; MFI, mean fluorescence intensity; PD-L1, Programmed death-ligand 1; CMTM6, CKLF-like MARVEL transmembrane domain containing 6; BafA1, Bafilomycin A1; ns, statistically not significant.

PD-L1 mRNA was not downregulated in AAV monocytes, indicating a regulation on the post-transcriptional level. Breakdown and degradation of surface PD-L1 have been reported to occur in lysosomes (33). Thus, we hypothesized that lysosomal degradation contributes to PD-L1 deficiency in AAV monocytes. Indeed, Bafilomycin A1 treatment that inhibits lysosomal function restored PD-L1 expression on AAV monocytes without affecting PD-L1 expression on HC monocytes (**Figures 5A, B**). A similar effect was observed when AAV monocytes were treated with chloroquine (**Supplementary Figure 7B**). One of the functions of chloroquine is the inhibition of lysosomes.

The protein chemokine-like factor-like MARVEL transmembrane domain containing family member 6 (CMTM6) emerged as a master regulator of the PD-L1 protein pool by preventing PD-L1 from being targeted for lysosome-mediated degradation (25, 26). CMTM6 protein levels were reduced in circulating AAV monocytes (**Supplementary Figure 5B**) as wells as after activation with IFNγ (**Figures 5C, D**). CMTM6 protein levels correlated with PD-L1 expression (**Figure 5E**). Lower expression of CMTM6 in AAV monocytes corresponded to a reduction in *CMTM6* mRNA (**Figure 5F**).

Collectively, these data demonstrated that CMTM6 is reduced in AAV monocytes, thus facilitating lysosomal degradation of PD-L1. Blocking lysosomal function restored their capacity to upregulate PD-L1.

### PD-L1/CMTM6-Defect Is Preserved in Monocyte-Derived Macrophages

**Figure 6:**
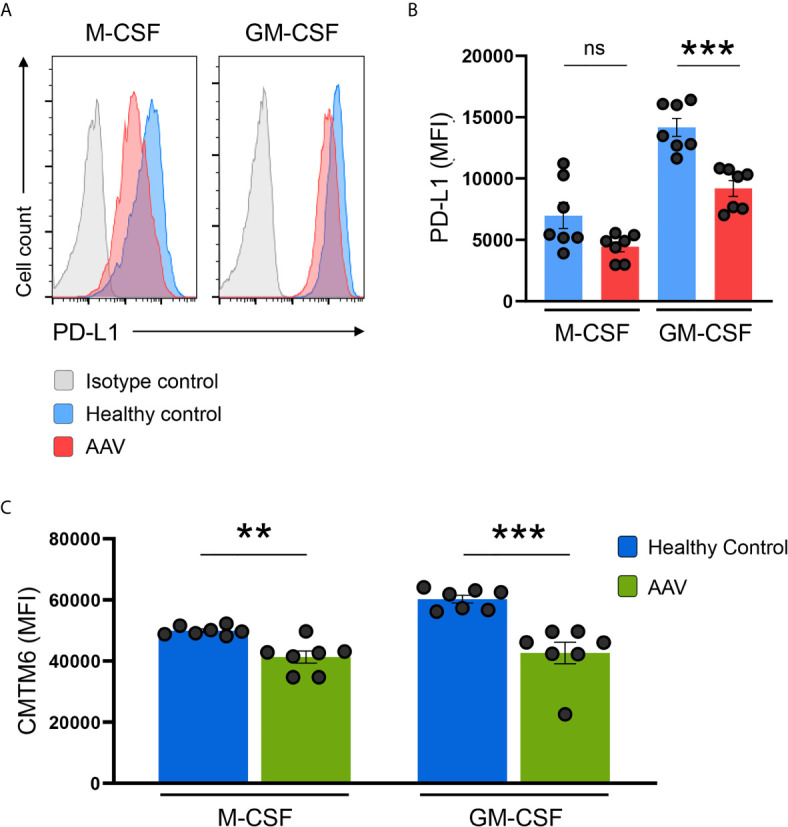
PD-L1/CMTM6-defect is passed to monocyte-derived macrophages. **(A)** Representative histograms of PD-L1 expression (MFI) on monocyte-derived macrophages after differentiation with M-CSF or GM-CSF for 6 days. **(B)** Summarizing scatter dot plot presenting results from experiments with cells from HC or AAV patients (n=7 each group). **(C)** Expression of CMTM6 protein in monocyte-derived macrophages from HC or AAV patients (n=7 each group). Mann-Whitney test **(B, C)** was applied. **P<0.01; ***P<0.001. Bar graph shows mean ± SEM. Bar graph shows mean ± SEM. AAV, ANCA-associated vasculitis; HC, healthy control donors; MFI, mean fluorescence intensity; PD-L1, Programmed death-ligand 1; M-CSF, macrophage colony-stimulating factor; GM-CSF, Granulocyte-macrophage colony-stimulating factor; ns, statistically not significant.

Monocytes can alter their phenotype based on environmental signals, e.g. they can differentiate into monocyte-derived macrophages. To study whether the defect in PD-L1 and CMTM6 expression was present also in macrophages, we differentiated monocytes with M-CSF or GM-CSF as previously described (29). After 6 days of differentiation, GM-CSF was a more potent inducer of PD-L1 than M-CSF. Monocyte-derived macrophages from AAV patients had lower expression of PD-L1 after differentiation with GM-CSF; after M-CSF differentiation there was a trend for lower PD-L1 expression in patients (**Figures 6A, B**) and CMTM6 (**Figure 6C**) compared to macrophages from HC donors. This suggests that the underlying defect for PD-L1 deficiency is imprinted to monocytes from AAV patients and carried on to corresponding cells once they differentiate.

### Monocytes From AAV Patients Show an Enhanced Stimulatory Capacity

**Figure 7:**
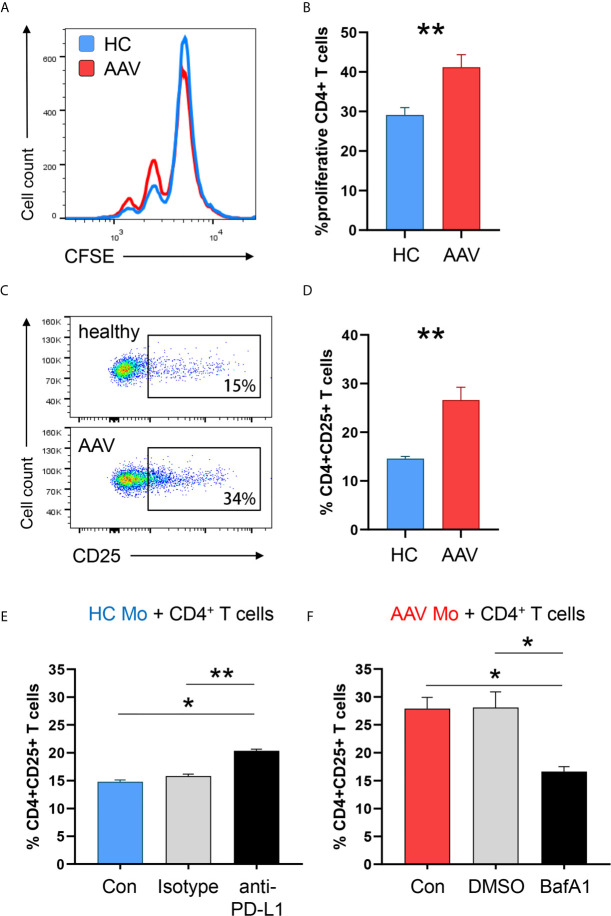
PD-L1^lo^ monocytes from AAV patients show enhanced stimulatory capacity. Monocytes from healthy or AAV donors were pretreated with IFNγ for 24h. Then, their capacity to stimulate T cells was probed by coculturing them with CD4+ T cells from healthy donors (ratio monocytes/T cells 1:3). T-cell proliferation was determined through CFSE dilution and T-cell activation was quantified by the frequency of CD4^+^CD25^+^ T cells. **(A)** Proliferation of CD4+ T cells was measured by flow cytometry after 5 days of co-culture. Representative histograms of CSFE expression. **(B)** Frequencies of proliferating CD4+ T cells when cocultured with either HC or AAV patient-derived monocytes (n=8 each group). **(C)** Activated CD4^+^CD25^+^ T cells quantified by flow cytometry after 48 h. **(D)** Percentage of activated CD4+ T cells after coculture (n=6 each group). **(E)** Co-culture with monocytes from healthy donors was performed in the presence of anti-PD-L1 antibodies or isotype control. Frequencies of activated CD4^+^CD25^+^ T cells from 6 independent experiments (isotype n=3) were measured after 48 h by flow cytometry. **(F)** Co-culture with monocytes from AAV patients was performed after monocytes were pre-treated with the lysosomal inhibitor BafA1 (20nM) or DMSO vector control. Frequencies of activated CD4^+^CD25^+^ T cells from 6 independent experiments (DMSO n=3) were measured after 48 h by flow cytometry. Mann-Whitney test **(B)**, unpaired t-test **(D)** and Kruskal-Wallis test with Dunn’s multiple comparisons test **(E, F)** were applied. *P<0.05; **P<0.01. Bar graph shows mean ± SEM. Bar graph shows mean ± SEM. AAV, ANCA-associated vasculitis; HC, healthy control donors; MFI, mean fluorescence intensity; PD-L1, Programmed death-ligand 1; M-CSF, macrophage colony-stimulating factor; GM-CSF, Granulocyte-macrophage colony-stimulating factor.

To examine the ability of PD-L1^lo^ AAV monocytes to stimulate T cells, we measured monocyte-induced T cell activation and expansion (in HC-derived T cells) by adapting a previously published co-culture model (16). AAV monocytes and HC monocytes were cultured with CFSE-labeled CD4^+^ T cells, after 5 days frequencies of dividing CD4^+^ T cells were measured. Frequencies of proliferating CD4^+^ T cells were higher in co-cultures with AAV monocytes (**Figures 7A, B**). Additionally, AAV monocytes enhanced early T cell activation as measured by the frequency of CD4^+^ CD25^+^ T cells after 48 hours (**Figures 7C, D**).

To understand whether the enhanced activation of T cells primed by monocytes is directly attributed to PD-L1 expression, anti–PD-L1 antibodies were added to co-cultures with monocytes from HC donors. Blocking PD-L1 on HC monocytes simulated PD-L1 deficiency and increased CD4^+^ T-cell activation (**Figure 7E**), confirming published data (16). To further elucidate whether blockade of PD-L1 degradation in lysosomes normalizes the hyperstimulatory behavior of patient-derived cells, AAV monocytes were pre-treated with the lysosomal inhibitor Bafilomycin A1. Inhibiting lysosome function in AAV monocytes restored their ability to balance immune cell interaction with T cells, resulting in less CD4^+^ T cell activation (**Figure 7F**).

In essence, PD-L1^lo^ monocytes from AAV patients cause enhanced stimulation of CD4^+^ T cells. Targeting lysosomal degradation of PD-L1 in AAV monocytes corrected this phenotype.

## Discussion

The development of autoimmune small-vessel vasculitis has been observed after checkpoint inhibitor therapy (21–24), indicating a relevant role of immune checkpoint molecules in the disease process. However, no molecular alterations of such molecules in the immune system of patients with AAV have been reported so far. In this study, we found that monocytes from AAV patients show a defect in presenting the immunoinhibitory checkpoint PD-L1 leading to enhanced stimulation of T cells.

Physiologically, the co-inhibitory ligand PD-L1 shows limited expression on circulating monocytes (30) and in normal tissues (11). Induction of PD-L1 on antigen-presenting cells occurs rapidly after cell activation to instigate a negative feedback loop thereby preventing overactivation of the adaptive immune system, e.g. of CD4^+^ T cells. Failure of PD-L1 induction promotes autoimmunity as reported for giant cell arteritis, an immune-mediated large vessel vasculitis, in which PD-L1-deficient dendritic cells facilitate inflammatory vascular damage (16). In AAV lesions, monocyte infiltration is a characteristic hallmark and vascular infiltrates show a predominance of monocytes and monocyte-derived macrophages. When entering tissue sites, monocytes encounter a multitude of pro- and anti-inflammatory stimuli and their response determines whether inflammation resolves or amplifies. In the case of AAV, PD-L1^lo^ monocytes may disturb the immunomodulatory PD-L1/PD-1 axis, thereby contributing to enhanced activation of CD4^+^ T cells and, thus, consolidating the chronic inflammatory process.

PD-L1 protein deficiency was not accompanied by decreased *PD-L1* mRNA transcripts in AAV monocytes, which indicated a regulation on the post-transcriptional level. Cleavage of PD-L1 by metalloproteinases (MMP) has been reported (34, 35) with tumor-derived MMP-13 potently degrading PD-L1 (36). In our experiments, inhibition of MMP-13 did not affect PD-L1 expression of monocytes (data not shown). Lysosomes are the cell’s degradation center and are responsible for the breakdown of proteins, polysaccharides, and complex lipids (37). Recently, two independent reports identified CMTM6, a protein of previously unknown function, as a major regulator of the PD-L1 protein pool. CMTM6 co-localizes with PD-L1 and prevents PD-L1 from being targeted for lysosome-mediated degradation (25, 26). Blocking lysosomal function with a specific inhibitor corrected PD-L1 deficiency in AAV monocytes indicating increased lysosomal breakdown of PD-L1 due to low levels of CMTM6. A similar effect was observed when AAV monocytes were treated with chloroquine. Chloroquine and hydroxychloroquine (HCQ) also impair lysosomal function but are less specific as they interfere with toll-like receptors and intracellular nucleic acid sensors (38). Interestingly, some groups report the successful use of HCQ in patients with AAV (39), and currently, a phase-II study is ongoing that evaluates HCQ in the treatment of AAV (HAVEN: Hydroxychloroquine in ANCA Vasculitis Evaluation, NCT04316494).

Emphasizing the role of monocyte PD-L1 expression in the disease course of AAV, higher ANCA titers correlated with lower numbers of circulating PD-L1+ monocytes in vasculitis patients. Moreover, their diminished capacity to present PD-L1 upon cell activation predicted higher ANCA titers, higher CRP serum concentrations, and active disease.

An unexpected finding of this study was the influence of colony-stimulating factors (CSFs) on PD-L1 expression. Higher PD-L1 surface expression was observed when monocytes were differentiated to macrophages with GM-CSF instead of M-CSF. In the literature, only one report describes the effect of GM-CSF on PD-L1, showing that tumor-derived GM-CSF induced PD-L1 expression in neutrophils (40). PD-L1 induction by GM-CSF could be part of a negative feedback loop to prevent uncontrolled inflammation sparked by more pro-inflammatory GM-CSF-differentiated macrophages. Alternatively, lower PD-L1 expression by M-CSF may reflect the greater lysosomal activity of M-CSF-derived macrophages (41). Serum levels of M-CSF are increased in AAV patients with active nephritis (42) and renal M-CSF production is upregulated in vasculitic glomeruli where it associates with local macrophage proliferation (43), suggesting an M-CSF-skewed macrophage phenotype in renal disease in AAV.

The design of this study bears limitations. The majority of AAV patients receive immunosuppressive therapy, which could alter any kind of read-out. AAV is a rare disease and often presents with severe symptoms making an immediate start of therapy inevitable. Our subgroup analysis as well as experiments testing the direct effects of cortisone on PD-L1 expression did not indicate evidence for a medication bias towards lower PD-L1 expression. Although we cannot exclude other factors in the natural course of the disease, the correlation of PD-L1 with markers of inflammation and disease activity suggests that low PD-L1 expression on monocytes is associated with disease activity.

Another limitation is the use of RA monocytes as disease control. To exclude that chronic inflammation lowers PD-L1 expression, we tested monocytes from RA patients, a typical chronic-inflammatory disease. It could be argued that inflammation in those patients is localized mainly in the joints and not in the vessel wall. Still, RA patients do have a component of systemic inflammation, which results in their increased cardiovascular risk (44) - as such a vascular inflammatory process. Further studies in other autoimmune disease would be needed to clarify whether low PD-L1 expression is specific for vasculitis. In connective tissue disease, in SLE for example, contradictory results have been reported with one group finding low PD-L1 expression on monocytes in a pediatric cohort (45) and another study reporting upregulation of PD-L1 on SLE monocytes (46).

In summary, this study identified a defective immunoinhibitory PD-L1 checkpoint on monocytes and monocyte-derived macrophages from patients with AAV. CMTM6-deficient vasculitic monocytes degrade PD-L1 in lysosomes, thus providing insufficient negative signaling to CD4^+^ cells, fostering the development of highly activated T cells in patients with autoimmune small-vessel vasculitis. Correcting this defect in monocytes by targeting lysosomal function may be a promising novel strategy to treat AAV, especially to maintain remission.

## Data Availability Statement

The raw data supporting the conclusions of this article will be made available by the authors, without undue reservation.

## Ethics Statement

The studies involving human participants were reviewed and approved by the Institutional Review Board (Ek 218/20, Ek 383/19) of the University of Freiburg. The patients/participants provided their written informed consent to participate in this study.

## Author Contributions

MZ, JT, and RV conceived the study. MZ performed experiments. MZ and NV analyzed data. BS contributed technical expertise. NC and MR enrolled patients and oversaw patient recruitment. MZ, NV, JT, and RV wrote the manuscript. All authors contributed to the article and approved the submitted version.

## Funding

This study was supported by the “Else-Kröner-Fresenius-Stiftung” (NAKSYS– 016_Kolleg.03) providing a fellowship to MZ, and the European Regional Development Fund of the European Union (INTERREG V programs RARENET and PERSONALIS to RV and MR). MZ was funded by the Research Commission of the Faculty of Medicine, University of Freiburg. JT was supported by the Berta-Ottenstein-Programme for Advanced Clinician Scientists, Faculty of Medicine, University of Freiburg. NC was supported by the Ministry of Science, Research, and Arts Baden-Wurttemberg (Margarete von Wrangell Programme).

## Conflict of Interest

The authors declare that the research was conducted in the absence of any commercial or financial relationships that could be construed as a potential conflict of interest.
